# Graphene-Based Coatings for Surface Modification and Their Applications in Fixed Orthodontics: A Scoping Review

**DOI:** 10.3390/dj11120285

**Published:** 2023-12-11

**Authors:** Arturo Garrocho-Rangel, Juan Carlos Flores-Arriaga, Cristina Zamora-Soberón, Alan Martínez-Zumarán, Socorro Ruiz-Rodríguez, Amaury Pozos-Guillén

**Affiliations:** 1 Pediatric Dentistry Postgraduate Program, Faculty of Dentistry, University of San Luis Potosí, 2 Manuel Nava, Zona Universitaria, San Luis Potosí 78290, S.L.P., Mexico; arturo.garrocho@uaslp.mx (A.G.-R.); msruiz@uaslp.mx (S.R.-R.); 2 Orthodntics Postgraduate Program, Faculty of Dentistry, University of San Luis Potosí, 2 Manuel Nava, Zona Universitaria, San Luis Potosí 78290, S.L.P., Mexico; carlos.flores@uaslp.mx (J.C.F.-A.); acristina.25aczs@gmail.com (C.Z.-S.); alanzuma@uaslp.mx (A.M.-Z.)

**Keywords:** archwires, brackets, coating material, graphene, scoping review

## Abstract

Background: Surface coating technology can assist fixed appliances by reducing friction, improving antibacterial characteristics, and increasing corrosion resistance. The application of functional coatings composed of graphene onto the surfaces of orthodontic brackets and archwires has been shown to enhance their mechanical qualities. The objective of the current study was to carry out a scoping analysis of published recent evidence on the utilization of graphene as a covering material in metallic orthodontic accessories, such as brackets and archwires; Methods: A scoping review was undertaken following the PRISMA-ScR guidelines. PubMed, Embase, the Cochrane Library, Dentistry and Oral Science Source, and Google Scholar were searched between 2003 and 2023; Results: In total, 38 potential references were detected, from which 10 were selected for this review. These articles addressed the benefits of the application of graphene-oxide functional coatings onto the surface of archwires and brackets during fixed orthodontic treatment. Orthodontic graphene-oxide-based coatings provide improved surface characteristics (e.g., reduced friction and anticorrosive effects), antibacterial capabilities, and biocompatibility. These characteristics can increase the effectiveness of orthodontic therapy, improve patient comfort, and lower the likelihood of problems; Conclusion: Orthodontists should be aware of and comprehend the prerequisites for using graphene-oxide-coated archwires and brackets to fulfill needs throughout their clinical practice.

## 1. Introduction

Nanotechnology involves modifying matter at a size smaller than 100 nm to produce a variety of biomaterials with different characteristics and uses. The scientific community has recognized the merits of carbon-based nanomaterials since the identification of carbon nanotubes in 1991 and fullerene in 1985. The strongest and thinnest substance currently in use is graphene. It is made up of hexagonally packed 2D single/bilayer/multilayer sheets of sp2 hybridized carbon atoms that are less than 10 nm thick and bound together in a honeycomb-like lattice. A single carbon atom forms a strong bond with another three atoms inside each sheet, which are arranged at an angle of 120°, with an interatomic distance of 1.42 and a center-to-center distance of 2.46. The characteristics of graphene change as the number of sheets rises [1].

Novoselov and Geim [2] conducted the initial research on graphene in 2004. They claimed that graphene is the fundamental component of all graphitic carbon materials, including carbon nanotubes, nanoribbons, diamond, and graphite. Graphene possesses remarkable physicochemical, optical, electrical, and biocompatibility properties. It also has a high surface area with distinctive properties and superior mechanical characteristics, for what it is a very resistant biomaterial. Graphene has a 130 GPa fracture strength and Young’s modulus of 2.4 ± 0.4 and 2.0 ± 0.5 TPa for single- and bilayer sheet forms, respectively. Graphene has a transmittance of roughly 97.7% and is also a very lightweight material with excellent intrinsic mobility. Today, graphene can be found in a variety of sizes, forms, and compositions, as well as with a variety of subsequent functionalization options. Graphene sheets can be changed into their derivatives, for example, reduced graphene oxide (rGO), graphene oxide (GO), and pure graphene (PG), through chemical and physical alterations.

The potential biological applications of graphene have been studied in many ways, including scaffolds for drug and gene delivery (e.g., anti-cancer-targeted drugs), imaging agents for disease detection, biosensors, and bimolecular analysis [1]. Furthermore, the field of nanomedicine has become quite interested in their potential use as antibacterial agents [3,4]. When graphene is incorporated into diverse biomaterials for clinical uses, such as surgical sutures, these antibacterial qualities could be very helpful in preventing the growth of bacteria [5]. The physical and chemical properties of graphene, including the number of sheets, chemical functional groups, and surface charge density, are what determine its toxicity. The biocompatibility and toxicity of nanostructures made of graphene have been the subject of numerous reports, although they have not yet been thoroughly studied.

Dental implants, root canal treatments, restorative/conservative dentistry, guided periodontal tissues, bone regeneration, oral and maxillofacial surgery, prosthetics, and tissue engineering (e.g., scaffolds for carrying tricalcium phosphate, dental pulp, or periodontal ligament stem cells) have all been shown to benefit from the incorporation of graphene in dentistry [5,6]. In orthodontics, nanotechnology—including graphene—has been used to produce shape memory polymers and orthodontic bonding materials by coating brackets, archwires, elastomeric ligatures, and adhesives [7]. This is due to this previously mentioned biomaterial’s improved strength, decreased friction, and microbicidal characteristics [8]. Not only may they prevent the growth of biofilms and lower cariogenic bacterial activity, but they can also speed up orthodontic treatment by moving the teeth more efficiently [7].

Considering nanomaterials have improved mechanical, physicochemical, and antibacterial qualities, new advancements in nanotechnology have provided better options for both patients and orthodontists [6]. In orthodontics, controlled pressures are employed to trigger a biological reaction that moves the teeth. Despite the availability of numerous mechanical orthodontic systems, presently the most widespread technique continues to be sliding tubes or brackets over an archwire. The magnitude of force applied should be optimized to maximize tooth displacement while minimizing any negative consequences [9]. In order to improve treatment effectiveness, decrease anchorage consumption, decrease the occurrence of caries and periodontal disease, as well as enhance the safety and longevity of orthodontic attachments, it is imperative to diminish the level of friction between the archwire and brackets. Additionally, it is crucial to enhance the antibacterial characteristics of orthodontic appliances and augment their resistance to corrosion and wear through the application of surface coatings [10]. This can be implemented by creating a suitable additional layer on the surface of the metallic orthodontic components, brackets, and archwires and using various coatings on them to change the surface morphology, mechanical properties, and antibacterial properties [11]. Surface coating technology makes use of a variety of metals, polymers, and composite materials. This is currently a significant method for enhancing the effectiveness of fixed orthodontic appliances, enabling therapeutic advantages and benefits like better corrosion resistance, friction reduction, and antibacterial effects [10,12].

The objective of the current study was to perform a scoping assessment of recently published evidence regarding the utilization of graphene as a covering material in metallic orthodontic accessories, such as brackets and archwires. In addition, we aimed to summarize the best available information about the possible therapeutic benefits (e.g., antimicrobial properties against cariogenic bacteria, reduced friction between orthodontic wires and brackets, and anticorrosive effects) associated with this emerging coating biomaterial.

## 2. Materials and Methods

### 2.1. Design 

The current scoping review was conducted in accordance with the method suggested by Arksey and O’Malley [13], Levac et al. [14], and the Preferred Reporting Item for Systematic Reviews and Meta-analysis guidelines for scoping reviews (PRISMA-ScR) [15]. According to Levac et al. [14], a scoping review includes the mapping and synthesis of the pertinent literature that already exists on a wide range of the fundamental ideas underlying a clinical topic of interest [16]. When a topic has not been thoroughly studied before, or when the literature exhibits a complex and varied nature that defies a more focused systematic review, this sort of review examines the primary sources and categories of evidence that are accessible [13,16]. Typically, a scoping review encompasses five stages: (i) the development of a precise research question, (ii) the identification and retrieval of pertinent studies, (iii) the process of selecting relevant studies, (iv) the extraction and organization of data, and (v) the compilation, synthesis, and presentation of findings.

To address and outline the search strategies, a focused question was structured based on the PICO acronym, as follows: in patients ongoing fixed orthodontics treatment (P), does the application of graphene coatings on orthodontic metallic adjuncts (I) represent any benefits and advantages in terms of anti-cariogenic bacteria activity, less friction on wire-brackets, and enhanced anticorrosion resistance (O) compared with non-coated surfaces (C)?

### 2.2. Article Selection Criteria

The present scoping review incorporated articles that satisfied the following criteria:

1. Original articles published in the English language concerning the effectiveness of the different applications of graphene coatings in metallic orthodontic archwires and brackets.
2. Comparative randomized/non-randomized controlled clinical trials.
3. Observational studies (case–control and cohorts).
4. In vitro studies.

Narrative reviews, case reports/series, abstracts, preliminary studies, conference proceedings, editorials, letters to the editor, expert comments, and gray literature were not considered.

### 2.3. Literature Search Strategies

A comprehensive search was conducted across five electronic databases (PubMed, Embase, the Cochrane Library, Dentistry and Oral Science Source, and Google Scholar). The purpose of this search was to ensure the inclusion of all pertinent primary studies published within the past two decades, specifically from January 2003 to September 2023. During the initial search, the following main MeSH and keywords were combined: “graphene oxide” AND “coating” AND “fixed orthodontics” AND “functional surface modification”, with the limitation of human randomized clinical trials (RCTs) and observational studies, written in the English language. This search process was developed for PubMed and employed a combination of controlled vocabulary, synonyms, text words/search terms, Boolean operators, and different translations, as follows:

“graphene oxide”[All Fields] AND “coating”[All Fields] AND “fixed orthodontics”[All Fields] AND “functional surface modification”[All Fields] (“graphenated”[All Fields] OR “graphene s”[All Fields] OR “graphenes”[All Fields] OR “graphenic”[All Fields] OR “graphenized”[All Fields] OR “graphite”[MeSH Terms] OR “graphite”[All Fields] OR “graphene”[All Fields]) AND (“orthodontal”[All Fields] OR “orthodontic”[All Fields] OR “orthodontical”[All Fields] OR “orthodontically”[All Fields] OR “orthodontics”[MeSH Terms] OR “orthodontics”[All Fields])

Translations

graphene: “graphenated”[All Fields] OR “graphene’s”[All Fields] OR “graphenes”[All Fields] OR “graphenic”[All Fields] OR “graphenized”[All Fields] OR “graphite”[MeSH Terms] OR “graphite”[All Fields] OR “graphene”[All Fields]

orthodontics: “orthodontal”[All Fields] OR “orthodontic”[All Fields] OR “orthodontical”[All Fields] OR “orthodontically”[All Fields] OR “orthodontics”[MeSH Terms] OR “orthodontics”[All Fields]

The search strategy was then appropriately adapted for each of the other databases. In the first stage, titles and abstracts were meticulously screened by two previously trained, independent authors (J.C.F.-A. and C.Z.-S.) in a non-blinded standardized manner to determine articles that satisfy the established inclusion criteria. Following the elimination of duplicate entries, all chosen articles were obtained in their entirety in the form of full-text documents. A supplementary manual search was conducted on the bibliographies of the aforementioned articles. The inter-reviewer agreement Cohen’s kappa concordance was 0.93 for this selection process. Disagreements between reviewers were resolved by discussion and consensus through consultation with a third author (A.M.-Z.). After that, two other experienced reviewers ((S.R.-R. and A.P.-G.) independently carried out full-text comprehensive reading of potential pre-selected articles. For this stage, the inter-reviewer kappa agreement was 0.91. Again, any disagreement was solved with a third reviewer (A.G.-R.). When necessary, an attempt was made to contact the article’s authors to request further details or information on unclear or missing data, if available. Afterward, three reviewers (A.G.-R., S.R.-R. and A.P.-G.) evaluated the full texts for potentially relevant studies. Again, any discrepancy was resolved by discussion and consensus with a fourth author (J.C.F.-A.). To ensure consistency of the reviewing process, all bibliographic references were managed with the Covidence software (Melbourne Australia, https://www.covidence.org/), including citation identification and localization of potential additional cross-referenced articles. 

### 2.4. Data Extraction and Quality Assessment of Selected Studies

From each included article, relevant data were manually retrieved, using a pre-tested, standardized form, by two independent authors (C.Z.-S. and A.M.-Z.). The data-extraction form was consistently revised using an iterative method. The provided information encompassed the primary author’s name, publication year, country of origin, and study design; coating material and technique; purpose; and main findings. All of the authors then agreed to evaluate these findings in order to discuss them in this review. Evidence tables were also constructed. 

## 3. Results

**Figure 1:**
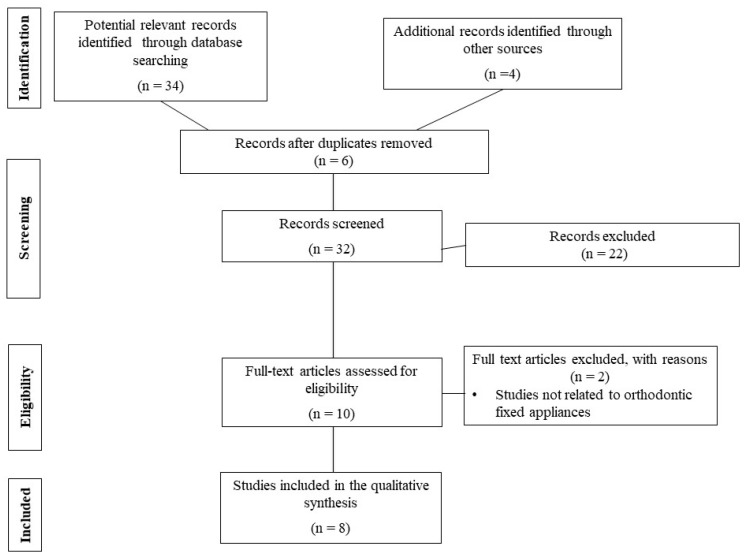
Flow diagram describing the selection process according to the Preferred Reporting Items for Systematic Reviews and Meta-Analyses (PRISMA) recommendations [15].

In the consulted electronic databases and the subsequent manual searches, 38 probable references were found. Afterward, 10 full-text papers were thoroughly examined for eligibility after title and abstract screening and duplicate removal. Finally, eight pertinent, representative, and instructive articles were included (no critical appraisal was conducted). The entire article search and selection process is described in a PRISMA flowchart (Figure 1). The number of found relevant records by electronic database was as follows: PubMed = 24, Embase = 7, the Cochrane Library = 1, Dentistry and Oral Science Source = 1, and Google Scholar = 1.

Considering the selected studies, seven of them were comparative in vitro trials [8,9,17,18,19,20,21] and one was an in vivo trial, conducted in mouse teeth [22]. No randomized or non-randomized clinical trials, case–control studies, or cohorts could be detected. The publication dates ranged from 2019 to 2023. Most studies on fixed orthodontic coatings were conducted on NiTi and stainless-steel alloys, employing three carbon-based biomaterials: graphene oxide alone, graphene oxide combined with silver nanoparticles, and graphene-sheet-embedded carbon. Five in vitro outcomes were mainly assessed: friction/lubricity between the archwire/bracket’s slot (coefficient of friction), corrosion/wear resistance, other tribological aspects, antibacterial properties, and oral tissue/cell biocompatibility. The principal characteristics, numerical data, and main findings/conclusions from each included article are summarized in Table 1 [8,9,17,18,19,20,21,22].

## 4. Discussion

Different archwires and brackets, which offer stabilization and may deliver various forms of orthodontic force, are incorporated into a fixed metallic orthodontic appliance. The alloys frequently utilized in fixed orthodontics for the production of brackets, wires, ligatures, bands, and other appliances are nickel-titanium (NiTi) and stainless steel [12]. The mechanical foundation of orthodontic therapy is based on the idea that tooth movement can convert accumulated elastic energy into mechanical work. Therefore, the utilization of a precise force system, complemented by metallic accessories such as orthodontic archwires, is important to provide optimal control over tooth movement. [8]. Orthodontic archwires are impacted by a variety of factors in the oral cavity environment, such as saliva flow, ingested liquids and food, temperature changes, tooth brushing, masticatory forces, and archwires sliding along the brackets [10]. As a consequence, plaque/biofilm accumulation, archwire/bracket friction or biocorrosion processes, and other inconveniences (e.g., the release of metal ions, which can act as allergens) may take place. In light of these considerations, recent studies have been undertaken to devise a range of approaches, such as coatings and surface treatments, aimed at enhancing the suitability of these materials for fixed orthodontic purposes. Specifically, the focus has primarily been on archwires and brackets, with the aim of addressing the aforementioned limitations. [12]. These technologies aim to change the surface morphology, mechanical characteristics, and biological properties of orthodontic appliances. In order to enable therapeutic effects, including friction reduction, antibacterial properties, and corrosion resistance, this is currently a potentially advantageous system to improve the functionality of the fixed appliance technique [10].

In order to enhance the surface qualities of fixed orthodontic appliances, some graphene coating processes and materials have been developed, according to Arango et al. [12] and Arici et al. [9]. For instance, NiTi wires have been painted using the electrostatic powder method [17,18,21]. On the other hand, orthodontic archwires have been coated using a different method of coating called physical vapor deposition [8]. Another method for coating metallic orthodontic materials is radio frequency (RF) magnetron sputtering [9,19]. By blasting a solid cathode (target) with positive ions from an inert gas discharge, this method removes surface atoms from the target and deposits the removed atoms on the surface to create a thin coating [19]. RF magnetron sputtering is an effective technique because of its inherent versatility, low-temperature thin film deposition, and uniform surface coating. Using this technique, better antiadhesive and antibacterial properties are provided [9]. Until now, these methodologies have primarily been employed in vitro to further assess the behavior of coatings, the biological features of coated substrates, and the mechanical attributes of both coatings and substrates [10]. Nevertheless, certain issues pertaining to coatings have been documented, primarily encompassing the delamination or erosion of these coatings [10]. 

The current scoping review intended to thoroughly review the most recent evidence about the above-mentioned potential properties of graphene-oxide functional coatings applied on the surface of fixed orthodontic metallic appliances (e.g., archwires and brackets): (i) reduced friction, (ii) anticorrosive effects, and (iii) anti-cariogenic bacteria activity. So, the present section will be focused on these three principal issues.

### 4.1. Reduction in Friction between Archwires and Brackets

Friction is described by Indumathi et al. [11] as “the resisting force tangential to the common boundaries between two or more bodies when under the action of an external force, one body moves or tends to move relative to the surface of the other”. One of the most crucial aspects of orthodontic therapy is the sliding friction coefficient, which includes both static and kinetic friction between the archwire and bracket during tooth movement [8]. Archwires with a round cross-section generate low forces, low friction, and surface wear into the bracket slot. Wires with a rectangular cross-section induce higher forces and, due to their complete adjustment to the bracket slot, provide full control of tooth movement, but evidently with increased friction and wear [23]. Only when the applied forces sufficiently outweigh the friction at the bracket slot/archwire interface will there be a biological tissue response in fixed orthodontics, leading to tooth movement [11]. Increased friction forces result in a reduction in the effective orthodontic force operating on the teeth, which in turn causes patient discomfort and pain, as well as damage to the teeth and periodontal tissues and a lengthier treatment time [8]. According to some studies, friction during orthodontic treatment can counterbalance 12–60% of the orthodontic force, decreasing the effectiveness of tooth movement and increasing anchoring consumption, both of which are harmful to the treatment [11,19]. In particular, a variety of nanocoatings have been developed to enhance the wear and friction qualities of the sliding contacts between orthodontic archwires and brackets [8,9,11]. 

Nonmetals, including carbon-based compounds like graphene, are considered to be relatively new and innovative materials for coating applications. Coatings that are based on graphene have demonstrated favorable characteristics in terms of both durability and biocompatibility. The application of this coating effectively reduces both static and dynamic friction by filling the surface grooves of the appliances and forming a lubricating layer [10]. Graphene coatings exhibit exceptional properties, including low friction coefficients, exceptional surface hardness, great resistance to wear, chemical inertness, and favorable biocompatibility [19]. The reduction in ligation force and subsequent decrease in friction achieved by these approaches has proven to be beneficial. However, it is important to note that the decreased contact between the wire and the bracket poses a challenge in terms of controlling orthodontic tooth movement [20,21]. Furthermore, it has been shown that the friction experienced between the archwire and the bracket when a rigid heavy wire is employed at a high angulation is similar to that seen with ordinary brackets [11]. Graphene oxide and graphene-sheet-embedded carbon (GSEC) are two compounds that are frequently employed as orthodontic coatings. NiTi alloy’s coefficient of friction can be decreased by applying graphene oxide coatings [17,20], and this coefficient can be further decreased by adding silver nanoparticles [17]. The GSEC surface coating of stainless steel archwires decreased the coefficient of friction to 0.12 in artificial saliva [19]. Wang et al. [8] have developed diverse techniques to make the friction on the surface of GSEC coatings even lower, reaching levels of less than 0.10. They found that the thickness of the coating increased as the GSEC film deposition time also increased, exhibiting very good friction reduction for up to one month [10]. In addition, Dai et al. [21] assessed the anti-frictional effects of a coating composed of graphene oxide (in two sizes, small and large) and silver nanoparticles applied on a NiTi substrate compared with non-coated or bare NiTi. They concluded that the experimental coating reached a markedly faster coefficient of friction regarding bare NiTi, with more stability, lubricity, and increased wear resistance, particularly in the small-graphene and silver coating group. 

### 4.2. Corrosion Resistance

Corrosivity and ion release are linked to the prolonged use of orthodontic wires and brackets in the oral cavity environment. Therefore, these materials ought to be immune to corrosion, stop ion leakage, and avoid causing allergic reactions. Since cytotoxicity and biocompatibility are linked to the corrosion process outcomes, corrosion behavior is frequently one of the main characteristics of metallic materials [21]. According to Martín-Cameán et al. [24], corrosion is a continuous process of degradation that occurs in the oral environment as a result of large changes in variables like pH, temperature, and microbial and salivary components. The corrosion of nickel-titanium (NiTi) archwires presents notable challenges in terms of biocompatibility due to their elevated nickel content, ranging from 47% to 50% [24]. The literature has documented that the introduction of nickel atoms into the oral cavity during clinical procedures has been associated with a range of negative responses, including minor hypersensitivity reactions as well as severe cytotoxic, mutagenic, and carcinogenic alterations [24]. Additionally, corrosion products can slow the movement of the teeth and raise the frictional coefficients between wire brackets. In addition, hydrogen ions can enter the NiTi alloy at an acidic pH and produce brittle titanium hydrides, which can lead to wire fracture. The sensitivity to corrosion is also known to be increased by fluoride ions found in a variety of anti-caries products [21,25]. Graphene is valued for its excellent mechanical properties in orthodontic material applications. There is active research on corrosion-resistant graphene oxide coatings; however, it is yet unclear how successful they are. Regarding this, a typical issue with corrosion-resistant graphene coatings is that long-term trials have not verified their longevity [10].

When applied to the surface of NiTi alloys by electrophoretic deposition, a technique created by Srimaneepong et al. [18], the graphene oxide/silver coatings showed reduced corrosion rates compared with those for uncoated NiTi alloys. This result suggests that the corrosion resistance of NiTi alloys can be improved by these graphene oxide coatings. Additionally, graphene oxide can result in NiTi archwires which are more resistant to wear. The surface of uncoated NiTi archwires produced numerous surface grooves as a result of wear, according to Dai et al. [20], but coated archwires produced relatively fewer grooves with a smaller width. Additionally, compared to concentrations of 0.5 or 5 mg/mL, a 2 mg/mL concentration of a graphene oxide coating produced less wear and a smoother surface after being subjected to mechanical friction. This proves that coating with graphene oxide at a 2 mg/mL concentration significantly improves wear resistance. Another type of graphene used as a surface coating with strong wear resistance is graphene-oxide-sheet-embedded-carbon (GOSEC). Pan et al. [19] coated stainless steel archwires with GSEC. After being subjected to friction, these archwires showed much fewer surface wear marks than non-coated archwires. On the other hand, the wear rate can reach a minimum of 0.11 × 10^−6^ mm^3^/Nm, according to Wang et al. [8]. In a recent report, Dai et al. [21] used pulse electrodeposition to create graphene oxide/silver nanocomposite coatings on the surface of NiTi alloys, utilizing small- and large-sized graphene oxide. Both coatings effectively increased the NiTi alloy’s corrosion resistance by filling in gaps and increasing the coating’s compactness. However, the corrosion resistance of small coatings was significantly superior to that of large coatings.

### 4.3. Antibacterial Properties

Microorganisms that appear and stick to the surfaces of fixed orthodontic appliances might lead to serious issues like enamel demineralization, white spot lesions, and biofilm-induced gingivitis. These metallic components are plaque-retentive niches, which hinder effective oral hygiene and increase biofilm retention [10]. Applying antimicrobial coatings, which prevent the growth of cariogenic microorganisms or protect against them, has therefore gained popularity recently. The first method is to use chemical or physical alterations to produce an antiadhesive surface that guards against bacterial adhesion and biofilm formation. The second is to develop coatings that release antibacterial substances. As a result, potential toxicity and resistance development are diminished. The third method is the application of microbicidal surface coatings with integrated antibacterial agents to ensure a long-lasting impact.

According to the literature, only Dai et al. [20,21] have conducted research on the antibacterial characteristics of graphene-oxide-based material coatings on NiTi alloys. Their first study showed that although the antibacterial activities were statistically different, *Streptococcus mutans* survival was only 20% worse when the coating could not completely cover the NiTi substrate, in which the graphene oxide concentration was low. The antibacterial qualities were improved when the concentration of graphene oxide was raised, but the biocompatibility diminished. Based on the aforementioned findings, it is recommended to maintain an appropriate concentration range of graphene oxide during the production of these coatings. However, the optimal value for this concentration has yet to be determined. Furthermore, the establishment of the biocompatibility of graphene coatings with respect to the human body remains inconclusive [20]. In their second study [21], they evaluated the antibacterial properties of graphene oxide (small and large size)/silver nanoparticle coatings against *S. mutans*. The coating exhibited a notorious antibacterial activity, which can be explained by its potential to wrap and trap bacteria to limit nutrient absorption, pierce the cell membrane, and break the bacterial structure. This fact was especially observed with large graphene oxide sizes due to the fast release of silver from the coating (approximately 48 h). In this same study, a remarkable bactericidal power of the coatings was demonstrated by the 90% reduction in the CFU number of surface *S. mutans*. On the other hand, a sizable number of living bacteria adhered to the bare NiTi surface, while hardly any dead bacteria were seen. So, the antiadhesion capabilities of these coatings were demonstrated by the greatly reduced number of *S. mutans* and the practically complete death of bacteria on the graphene/silver coatings. Further, surface bacteria exhibited a flattened, wrinkled, or deformed shape and showed no division period.

## 5. Conclusions

The surfaces of fixed orthodontic equipment can be altered using a variety of biocompatible materials and methods. However, only a small number are now employed in clinical orthodontics, particularly due to practical considerations like reducing cariogenic bacterial adhesion and controlling friction and corrosion. Therefore, there is still a long way to go before implementing clinical applications.

Orthodontic graphene-oxide-based coatings with improved surface characteristics and antibacterial capabilities have demonstrated significant biocompatibility to prevent serious cytotoxic effects on oral tissues. These characteristics can increase the effectiveness of orthodontic therapy, improve patient comfort, and lower the likelihood of problems. The antimicrobial activity and environmental resistance of these surface coatings can be improved by adding additives, including silver nanoparticles. Graphene-oxide coating techniques require additional studies, which will soon allow for direct clinical application in the oral cavity. 

## Figures and Tables

**Table 1:** Characteristics of included studies.

First Author (Year) and Country	Study Type	Coated Material and Technique	Purpose(s)	Main Findings and Conclusions
Rokaya et al. (2019) Thailand [17]	In vitro study	- NiTi alloy. - Electrophoretic Deposition.	- To assess the mechanical and tribological characteristics of a NiTi alloy coated with graphene oxide (GO) and silver (Ag) nanoparticles.	- The application of a nanocomposite coating consisting of GO/AgNP on a NiTi alloy resulted in enhanced mechanical strength and a decreased friction coefficient. - The results of the Raman spectrum supported the nanocomposite-coated surface. The coating surface’s thickness, roughness, and Young’s modulus all increased when the coating period was extended. In comparison to untreated alloys, the coated NiTi alloy substrate showed a lower friction coefficient. - Additionally, the surface coating’s friction coefficient decreased coating thickness dependently.
Srimaneepong et al. (2020) Thailand [18]	In vitro study	- NiTi alloy. - Electrophoretic Deposition.	- Corrosion resistance properties of graphene oxide (GO) and graphene oxide/silver (GO/Ag) nanocomposite coatings when exposed to a 3.5% sodium chloride (NaCl) solution. - Bare (not coated) NiTi alloy served as the control group. - Viability of pulp fibroblasts, and the expression level of interleukins IL-6 and IL-8 in 1% fetal bovine serum.	- Both GO-coated NiTi and GO/Ag coating on the NiTi alloys showed better corrosion resistance, a lower rate of corrosion, and higher protection efficiency regarding the bare NiTi alloy. - The biocompatibility of the NiTi alloys that were coated was demonstrated with human pulp fibroblasts, resulting in an increase in the levels of IL-6 and IL-8.
Pan et al. (2021) China [19]	In vitro study	- Orthodontic stainless steel archwires. - Electron cyclotron resonance (ECR) plasma sputtering system.	- To deposit graphene-sheet-embedded carbon coatings (GSECs) on readily available material. - To improve the friction and wear performances of archwire-bracket sliding contacts. - Artificial saliva.	- The GSEC film-coated archwires showed low friction coefficients and high wear resistance when sliding against metallic brackets in surroundings with artificial saliva. - The low-friction properties of the coated GSEC film were maintained even after 30 days of immersion in synthetic saliva solution. - The exceptional friction and wear characteristics of the archwires coated with GSEC film have been attributed to the formation of a salivary adsorbed layer and a tribofilm containing abundant graphene sheets at the contact surfaces.
Arici et al. (2021) Turkey [9]	In vitro study	- Aluminum oxide (Al_2_O_3_), titanium nitride (TiN), or chromium nitride (CrN) coatings. - Radio frequency magnetron sputtering.	- To evaluate the changes in friction between coated orthodontic brackets and archwires. - To assess the resistance of coatings exposed to intraoral conditions.	- Coating of both the metallic bracket and NiTi archwire with Al_2_O_3_ significantly reduced the friction coefficients (*p* < 0.01). - When the bracket and stainless steel archwire were coated with Al_2_O_3_ and TiN, the friction coefficients were significantly decreased (0.207 and 0.372, respectively) than when the bracket–archwire combination was not coated (0.552; *p* < 0.01). - The friction, thermal, and brushing tests did not weaken the global quality of the Al_2_O_3_ coatings. - Some small areas of peeling were clearly observed in the TiN coatings, whereas larger areas of peeling were detected in the CrN coatings.
Wang et al. (2022) China [8]	In vitro study	- The utilization of a bespoke electron cyclotron resonance (ECR) plasma sputtering deposition device, operating in the presence of electron irradiation.	- To evaluate the fretting friction and wear behaviors of the carbon film-coated archwires running against stainless steel brackets in an artificial saliva environment. - A home-built reciprocating sliding tribometer was constructed.	- The creation of a parallel micro-groove texture on the bracket slot surfaces along with an increase in GSEC film thickness significantly contributed to stable and low friction coefficients of less than 0.10. - After sliding across the three-row micro-groove textured bracket 10,000 times in fretting tests, the GSEC film did not wear out on the archwire. In addition to having a low friction coefficient (0.05), the GSEC film also had a low wear rate (0.11 × 10^−6^ mm^3^/Nm). - The synergistic effects of the parallel micro-groove textures manufactured on the bracket slot surfaces and the GSEC lubrication layer placed on the archwires are responsible for the considerable low friction coefficient and high wear resistance of the archwire–bracket sliding contacts.
Jiao et al. (2022) China [22]	In vivo study (mouse teeth)	- GO gelatin was prepared by reducing GO with gelatin, according to a previously proposed method by the same authors, and stored in water with 1.0 mg/mL concentration at 4 °C.	- To evaluate the effects of a biocompatible gelatin-reduced GO on bone marrow stromal stem cells (BMSCs) for promoting bone remodeling, in the aspects of osteogenesis and angiogenesis. - To verify the impact of this model on accelerating orthodontic tooth movement.	- The acceleration of mouse orthodontic tooth movement was seen in the group that received local injections of GOG, exhibiting increased levels of osteoclastic bone resorption and neovascularization in comparison to the control group. - The mechanism of GOG in promoting bone remodeling was clarified, which indicated the promised application of GOG in accelerating orthodontic tooth movement.
Dai et al. (2022) China [20]	In vitro study	- Silane coupling method.	- To deposit GO nanocoatings of different concentrations on the surface of NiTi alloy archwires. - To assess corrosion resistance, friction performance, and antibacterial properties as a function of the GO concentration.	- Different concentrations of GO coatings reduced the tendency for corrosion in the synthetic saliva when compared to the NiTi substrate and improved lubricity and anti-*Streptococcus mutans* performance. When the GO concentration in the coating was low, the corrosion and friction resistances were both insufficient. - When the concentration was too high, the coating’s corrosion resistance and friction resistance were insufficient. - Cells cultivated with the GO-coated NiTi were more vulnerable to the damaging effects of oxidative stress.
Dai et al. (2023) China [21]	In vitro study	- Pulse electrodeposition.	- To assess the surface properties and biological effects of GO and Ag in nanoparticles of variable sizes to deposit nanocoatings on NiTi alloys.	- Small-sized GO/Ag nanoparticle coating decreased the coefficient of friction to 0.1, reduced the corrosion current density by ten times, and decreased the number of corrosive ions. - In the coating containing large-sized GO/Ag nanoparticles, a limited optimization in terms of friction and corrosion resistance was exhibited. - Both coatings demonstrated biocompatibility with L929 cells and exhibited adherence to human gingival fibroblast cells. Also, they maintained a consistent antibacterial activity against 90% of *S. mutans* over a period of seven days, achieved through sustained release. - The size of GO could regulate the mechanical and biological properties of GO/Ag coatings.

## Data Availability

No new data were created in this study. Data sharing is not applicable to this study.
